# Intracranial stenosis prediction using a small set of risk factors in the Tromsø Study

**DOI:** 10.1186/s12911-025-02896-x

**Published:** 2025-02-20

**Authors:** Luca Bernecker, Liv-Hege Johnsen, Torgil Riise Vangberg

**Affiliations:** 1 Department of Clinical Medicine, UiT-The Arctic University of Norway, Tromsø, Norway; 2 PET Imaging Center, University Hospital of North Norway, Tromsø, Norway; 3 Department of Radiology, University Hospital of North Norway, Tromsø, Norway

**Keywords:** Intracranial stenosis, Machine learning, Artificial intelligence, Prediction, Predictive values

## Abstract

Intracranial atherosclerotic stenosis (ICAS) refers to a narrowing of intracranial arteries due to plaque buildup on the inside of the vessel walls restricting blood flow. Early detection of ICAS is crucial to prevent serious consequences such as stroke. Here we apply three different machine learning methods, such as support vector machines, multi-layer perceptrons and Kolmogorov-Arnold Networks to predict ICAS according to sparse risk factors from blood lipids and demographic data, including smoking habits, age, sex, diabetes, blood pressure lowering and cholesterol-lowering drugs and high-density lipoprotein. We achieved similar performance on classification compared to modern detection algorithms for ICAS in TOF-MRA (time-of-flight magnetic resonance angiography). The prevalence of ICAS in the population is relatively low, which is often case in medicine. While in the medical research community, the issue of low prevalence is established, machine learning-based research in medicine often does not take into account a critical viewpoint of the prevalence in clinical settings of their methods. We showed that with a balanced training/test set an accuracy up to 81% was achievable, while with the inclusion of prevalence, the positive predictive value was at 19% to the prevalence data, changes the performance metrics. Therefore, we highlighted the discrepancy that can arise between the results reported by the models and their clinical relevance. Furthermore, the results demonstrate the predictive potential of limited risk factors, highlighting its potential contribution to a multi-modular classification algorithm based on MRAs.

**Supplementary Information:**

The online version contains supplementary material available at 10.1186/s12911-025-02896-x.

## Introduction

Intracranial atherosclerotic stenosis (ICAS) refers to a narrowing of intracranial arteries due to plaque buildup on the inside of the vessel walls restricting blood flow. ICAS is a risk factor for ischemic stroke [1, 2] and is also associated with cognitive deficits and dementia [3]. Autopsy studies revealed that 43-31.4% of fatal strokes patients had ICAS [4, 5] and risk of stroke in the territory of the stenotic artery was highest with severe stenosis > = 70% with a hazard ratio 2.03 and a 95% confidence interval of 1.29 to 3.22 [6], where the risk for stroke increases with an inadequate mean arterial pressure [7]. Estimates of population prevalences for ICAS range considerably from 3 to 12%, likely due to differences in diagnostic methods and/or population samples [8–11]. Studies on hospital samples report a considerably higher prevalence of ICAS, ranging from 9 to 65% [12]. Early detection of stenosis is important for effective interventions or treatment, but diagnosis is labor-intensive and requires highly-trained personnel. Automated pathology detection using machine learning (ML) has shown promising results for related neuroradiological applications, such as aneurysms detection [13], but for detecting ICAS, there has been modest progress, with only two published methods in the last four years [14, 15], both of which had insufficient performance to be a useful clinical tool. This discrepancy may be because the salient features of ICAS are more subtle than those of aneurysms.

One way of improving ML models for detecting ICAS is to enrich the images with relevant metadata, such as known risk factors for ICAS. This approach has, for example, improved classification for skin lesions [16] and cardiomegaly [17]. For this approach to succeed, the metadata must have predictive power to detect ICAS.

To lay the groundwork for ICAS detection methods that utilize both image and metadata, we explore how well common risk factors can predict ICAS. We test the performance of three different ML models for predicting ICAS on risk factors only. Using the recently developed Kolmogorov-Arnold Networks (KAN) [18] and two more conventional and well-established methods Support Vector Machine (SVM) and Multi-Layer-Perceptron (MLP) [19, 20]. We further demonstrate the importance of considering the prevalence of a condition or disease when reporting the classification accuracy. This is often overlooked, but it is essential to recognize that an 80% accuracy with an 80% sensitivity does not directly translate to an 80% chance of accurate prediction for positively diagnosed patients. Therefore, an interpretation of the model without accounting for the disease prevalence does not inform on the real-world performance of model [21]. To accurately determine an individual’s likelihood of actually having the disease, it is important also to consider the positive predictive value (PPV), which depends on the prevalence [22]. We illustrate how considering predictive values alongside accuracy metrics gives a more realistic evaluation of predictive algorithm performance.

## Materials and methods

### Ethics

The study was approved by the Regional Committee of Medical and Health Research Ethics Northern Norway (619939 REK-Nord) and carried out in accordance with guidelines at UiT The Arctic University of Norway. All participants gave written informed consent before participating in the study. The data used in the analysis can be obtained by contacting The Tromsø Study (tromsous@uit.no).

### Data

**Fig. 1:**
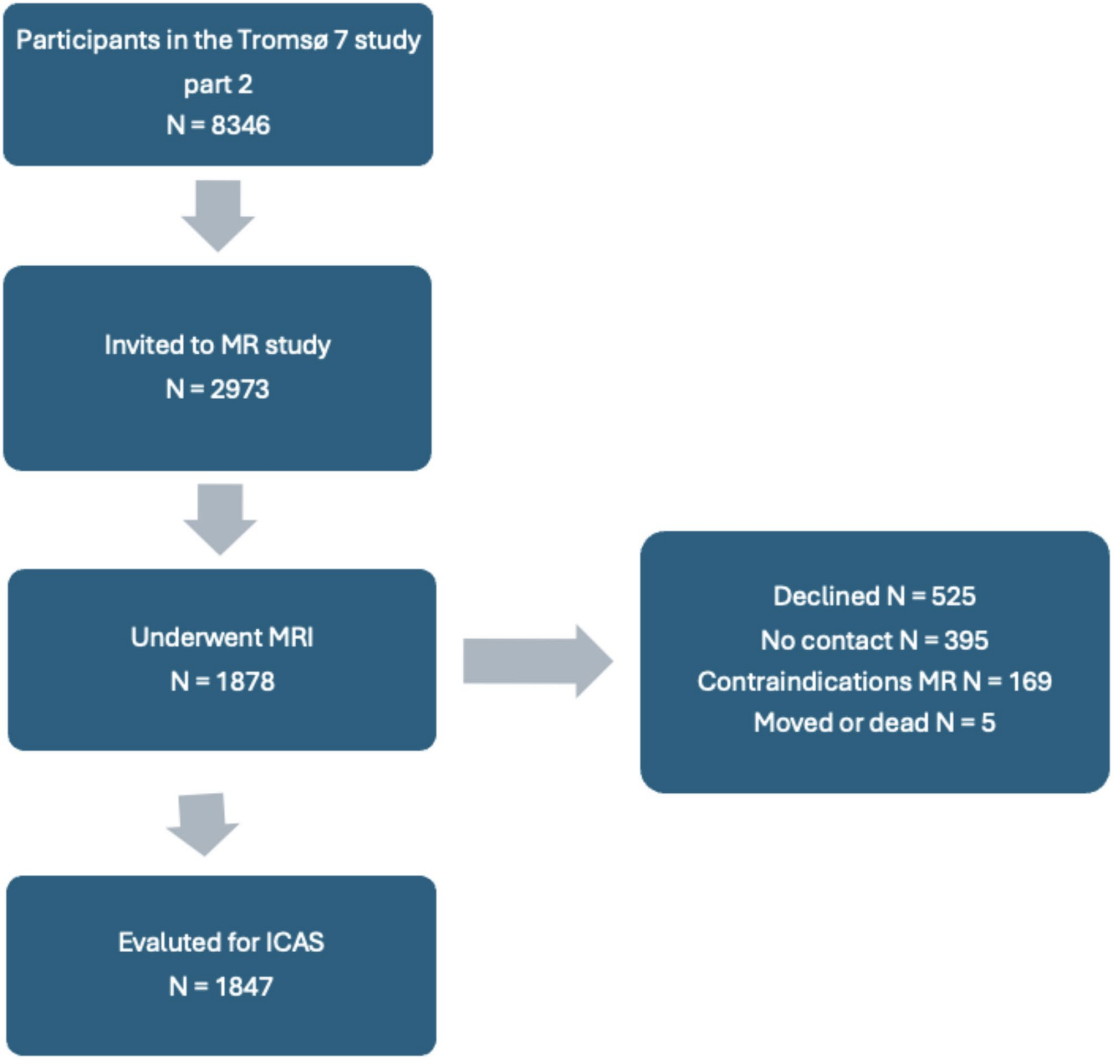
Flow chart of the selection of subjects from the seventh Tromsø study

We used data from the 7th Tromsø study, conducted between 2015 and 2016 [23]. The study collected demographic and health data from citizens 40 years or older in the Tromsø municipality. A subset of participants were recruited for a more detailed followup examination as seen in the flow chart in Fig. 1, and of these, 1878 received a cerebral MRI scan in 2016–2017, which included a 3D time-of-flight angiography sequence. In the present study, we used the 1847 cases graded for intracranial stenosis (the 31 excluded cases were due to missing data, insufficient image quality, intracranial artery disease, and withdrawal of consent) [8]. This manual grading was considered gold standard for the predictive model [8].

Stenosis was graded using the Warfarin-Aspirin Symptomatic Intracranial Disease method (WASID), expressed as the percentage occlusion in the stenotic artery relative to the proximal normal artery [24]. ICAS was defined as a 50% or greater degree of stenosis. For further details regarding the stenosis grading, we refer to [4].

### Predictive model

Recognizing the imbalanced data distribution with around 1700 non-ICAS cases to 111 ICAS cases, we employed a random under-sampling technique to create a balanced training dataset. This under-sampled data was split into 80 and 20% for training and testing purposes.

The three predictive models, SVM, MLP, and KAN were chosen to compare a classical machine learning algorithm that focuses on maximizing the margin between the two classes and compare them to the conventional neural network and recent advancements. The SVM was implemented with scikit-learn version 1.2.2 with a linear kernel and regularization parameter (*λ*) of 0.1. This was determined via a grid search hyperparameter optimization. MLP with TensorFlow 2.15.0, with two hidden layers, 100 neurons each, with a Rectified Linear Unit (ReLU) activation function and one output neuron with a sigmoid activation function. The algorithm used the Adams optimizer for 50 epochs with a learning rate of 0.03. KAN consisted of two hidden layers with 50 neurons and one output neuron and was built using a TensorFlow implementation of KAN (tfkan) from (https://github.com/ZPZhou-lab/tfkan). KAN was run 15 epochs with Adams optimizer with a learning rate of 0.005.

Due to the small number of observations, five-fold cross-validation was used, allowing for every stenosis case to be trained and tested in relation to the others. This results in a more robust evaluation that is less biased towards the selection of data [25]. Furthermore, the predictive model was evaluated three times as an undersampling method to account for the potentially skewed representation and overfitting of the non-afflicted population data due to the randomness of the sampling. In our model, we included well-established risk factors, that had an especially high odds ratio for ICAS age, sex, high-density lipoprotein (HDL) in mmol/L, cholesterol-lowering drugs, diabetes, blood pressure-lowering drugs, and smoking [8, 11, 26]. Smoking, diabetes, blood pressure medication and cholesterol lowering drugs were categorized as ”never”, ”previous”, and ”current”. Initial testing included blood pressure, but it was dropped due to the fact that the blood pressure medicine was a good indicator for blood pressure-related issues. We made the assumption that blood pressure and cholesterol-lowering drugs indicated a long exposure to the relevant risk factors. This long exposure is known to increase cardiovascular risk [27]. Blood pressure was used in an initial model, but resulted in a strong inequality between sensitivity and specificity and the reported risk factors gave the highest accuracy in the predictive models. The correlation matrix with all risk factors can be seen in the Supplementary material.

### Evaluation

The metrics used for the evaluation of the predictive models were sensitivity [28],

$$\:Sensitivity=\frac{TP}{TP+FN}$$

specificity,

$$\:Specificity=\frac{TN}{FP+TN}$$

and accuracy,

$$\:Accuracy=\frac{TP+TN}{TP+TN+FP+FN}$$

where *TP* stands for true positive, *TN* for true negative, *FP* for false positive, and *FN* for false negative. The predictive values can be calculated through Bayes theorem depending on accuracy and prevalence [22],

$$\:PPV = \frac{{PREV \cdot SENS}}{{PREV \cdot SENS + \left( {1 - PREV} \right) \cdot (1 - SPEC)}}$$

and

$$\:NPV = \frac{{\left( {1 - PREV} \right) \cdot SPEC}}{{\left( {1 - PREV} \right) \cdot SPEC + PREV \cdot (1 - SENS)}}$$

where *PREV* stands for prevalence, *SENS* for sensitivity, and *SPEC* for specificity.

## Results

The prevalence of ICAS in the population sample was 6%. Compared to the participants without ICAS, the ICAS group had a greater percentage of males, were older, more often hypertensive and diabetic, had higher BMI, and lower levels of HDL cholesterol (Table 1).

### Model performance

**Fig. 2:**
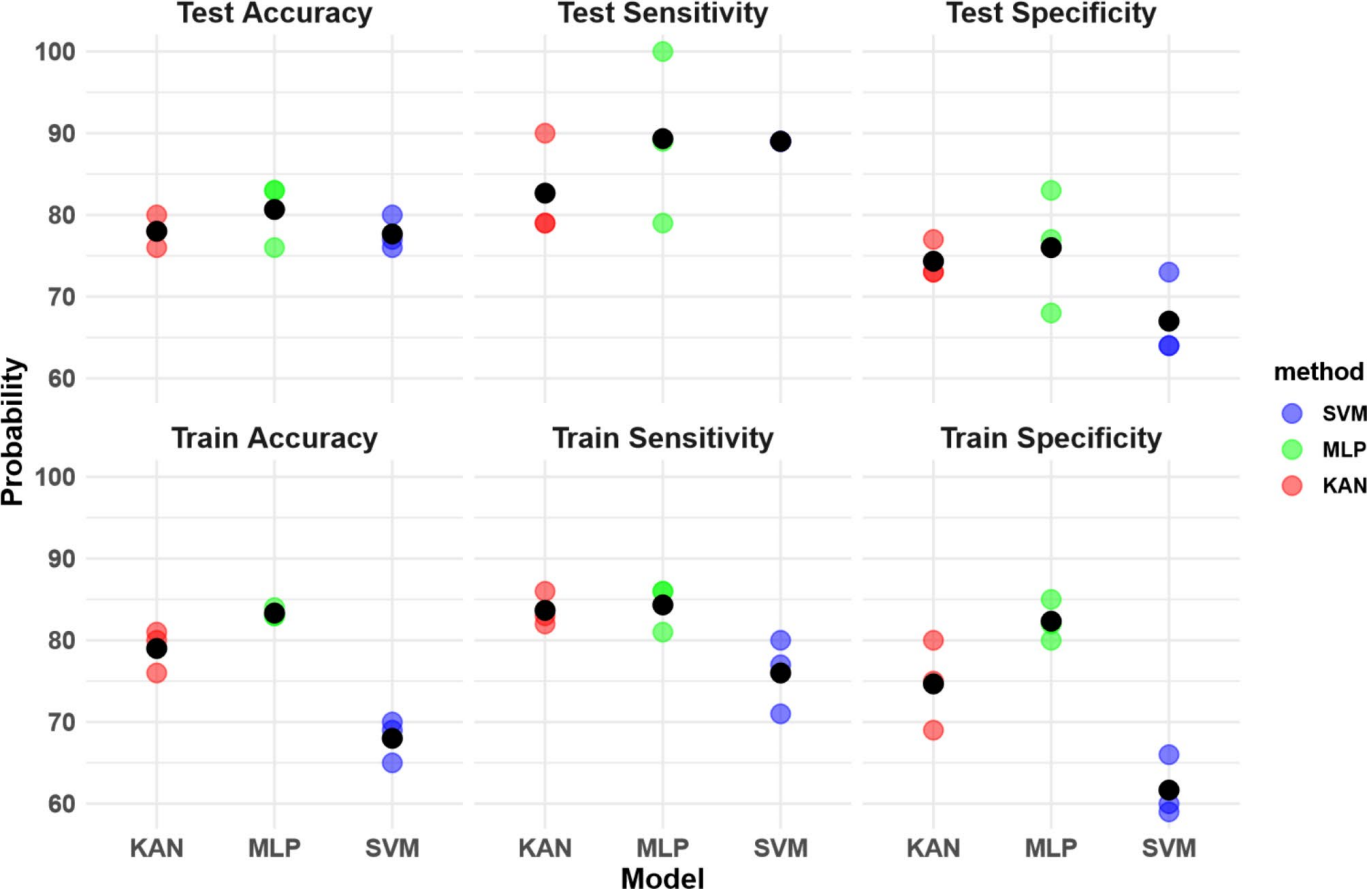
Comparison of performance metrics among Support Vector Machine (SVM), Multi-Layer Perceptron (MLP), and K-Adaptive Neurons (KAN) models. Performance metrics are train and test accuracy, sensitivity, and specificity for each model trained on random sampling states, the averages are given in black

**Fig. 3:**
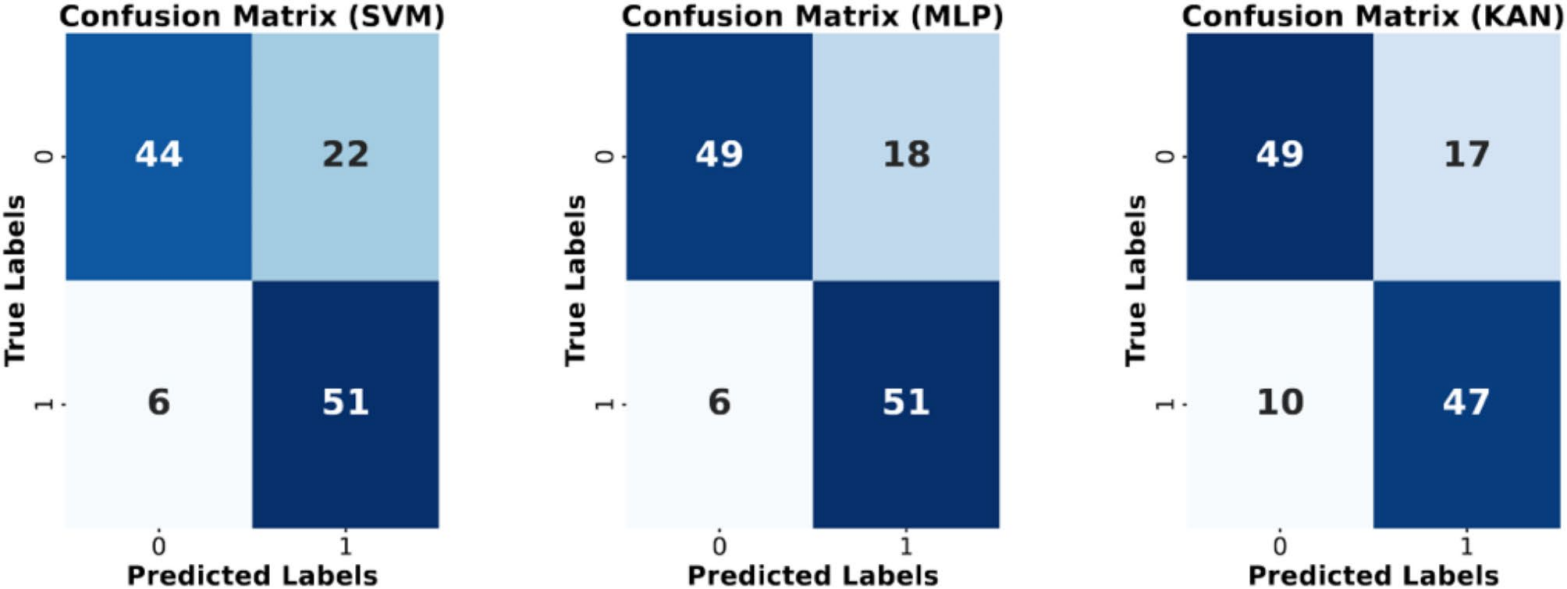
Confusion matrices representing the performance of Support Vector Machine (SVM), MultiLayer Perceptron (MLP), and K-Adaptive Neurons (KAN) models. Each matrix illustrates the classification results with actual labels on the y-axis and predicted labels on the x-axis. The numbers in each cell indicate the count of instances classified accordingly. Shades of blue represent the intensity of correct classifications, with darker shades indicating higher counts. The SVM, MLP, and KAN models are depicted in the first, second, and third matrices, respectively

The SVM, MLPs, and KAN models had a mean test accuracy of 78, 81, and 78%, with a specificity of 67, 76, and 74% and sensitivity of 89, 89, and 83%, respectively, as seen in Fig. 2. The difference between the MLP and KAN/SVM in accuracy is 3.0 percentage points. The algorithms were performed three independent times in succession. While under-sampling introduces a risk of features being over-represented due to randomness, our findings indicate a consistent distribution across the predictor space in all three instances of the random sampling. The detailed confusion matrix is presented in Fig. 3 and the histogram of predictions can be seen in the Supplementary material.

**Table 1:** Demographics and clinical data for pariticpants diagnosed with and without ICAS.

Variables	NO ICAS *N* = 1736	ICAS *N* = 111	*p*-value
Age	63.30 (10.53)	72.42(7.49)	< 0.001
Male sex, n (%)	797 (46%)	69 (62%)	< 0.001
Diastolic blood pressure, mmHg	75.08 (9.92)	76.15 (9.30)	0.2
Systolic blood pressure, mmHg	133.21 (20.50)	145.71 (20.51)	< 0.001
Serum LDL cholesterol, mmol/L	3.58 (1.01)	3.47(1.09)	0.3
Serum HDL cholesterol, mmol/L	1.64 (0.51)	1.45 (0.36)	< 0.001
Body mass index, kg/m2	27.04 (4.17)	28.15 (3.92)	0.001
Current smoker, Yes/No/Previous	218/650/850	18/38/55	0.5
Blood pressure lowering drugs, Yes/No/Previous	501/1160/50	60/42/7	< 0.001
Cholesterol lowering drugs, Yes/No/Previous	367/1252/75	57/47/5	< 0.001
Diabetes, Yes/No/Previous	89/1582/6	15/92/1	0.001

Abbreviations: low-density lipoprotein (LDL), high-density lipoprotein (HDL). Missing measurements (percentage relative to total): diastolic blood pressure 5 (0.3%), systolic blood pressure 4 (0.2%), serum LDL cholesterol 7 (0.4%), serum HDL cholesterol 7 (0.4%), body mass index 1 (0.1%), current smoker 18 (1.0%), blood pressure lowering drugs 27 (1.5%), cholesterol lowering drug 44 (2.4%), diabetes 62 (3.4%)

In Table 2, the positive and negative predictive values for the MLP model are 19% and 99%, respectively. While the sensitivity of 89% would indicate a high positive predictive value, the prevalence of 0.06 has a drastic impact on the predictive values. Assuming a prevalence of 50%, specificity and sensitivity have an equal impact on patient outcomes as predictive values.

**Table 2:** Positive predictive value (PPV) and negative preditive value (NPV) for the three models

Model	PPV (%)	NPV (%)	F1-score (%)
SVM	13	99	76
MLP	19	99	80
KAN	17	99	78

**Fig. 4:**
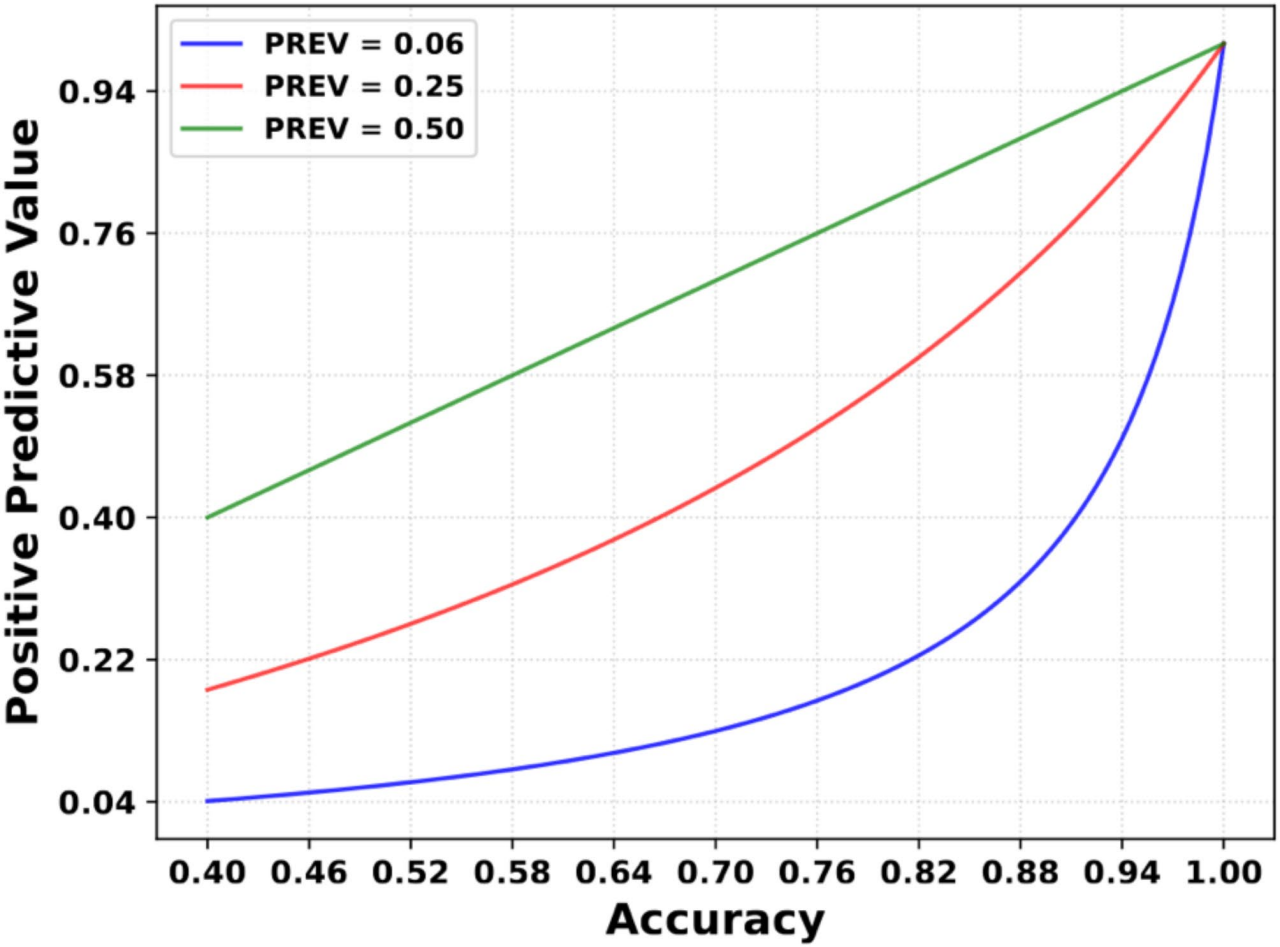
The positive predictive value (PPV) in terms of sensitivity and specificity in decimal representation. Both the values on the x-axis are set equal and portray the accuracy. The green line is how most models train on balanced data with a prevalence of 0.5

In Fig. 4 the PPV value is plotted with respect to sensitivity and specificity, which are set to be equal while maintaining a constant prevalence. The prevalence for the blue line reflects the population data in this study of 0.06, and the red line is the prevalence of ICAS for elderly Japanese men [29].

In the figure, it is evident that to attain an 80% certainty of correctly diagnosing a patient as positive, both sensitivity and specificity would have to be at least 95%. Although Fig. 4 is strictly only valid for the present study, similar trends hold for all clinical diagnostic methods where the disease prevalence is low.

## Discussion

Our main findings are twofold. First, we show that with basic clinical and demographic data, it is possible to achieve higher classification accuracy for ICAS than state-of-the-art detection algorithms using expensive imaging techniques. The limitation, of course, is that our method is unable to locate the stenosis. The high classification accuracy demonstrates the potential for using clinical data to enrich image data to further increase ICAS detection accuracy. Second, we demonstrate that the real-world accuracy of a classification algorithm is highly dependent on a disease’s prevalence.

Our results coincide with the promises of [18], where they introduce the idea that KANs perform with half the size and faster. The best performing model the MLP-based predictive model, has a test accuracy of 81.0% with a specificity of 76% and sensitivity of 89% by using a limited set of clinical and demographic data. In comparison, state of the art TOF-MRI based intracranial stenosis detection has a sensitivity for intracranial stenosis of 60.4% with a positive predictive value of 79.34%, dis-including absence segments of the Circle of Willis in the paper, where the ground truth is derived by manual inspections of the TOF-MRA by radiologists [14]. This illustrates that current pure TOF-MRI detection algorithms are not able to contribute to a clinical tool for classifying ICAS. By combining risk factors as well as images the algorithm obtains more diverse relevant data, which should result in a higher predictive power.

Furthermore, for a more advanced model, which may even evaluate the severity and risk factor of the ICAS without the need to image based data, Interleukin-6 and Lipoprotein-associated phospholipase A_2_ would allow the model a deeper understanding of ICAS and the causes of stroke [30, 31].

Second, despite our model exhibiting an 89% sensitivity, the PPV remains notably low at 19%. This prompts a critical examination of the actual impact of scientific predictive models, particularly those designed for low-prevalence conditions. An illustrative counterexample is the manual detection of intracranial stenosis [32]. The authors center their investigation on the detection within arterial segments, a distinction that complicates a direct comparison with patient testing methodologies. Despite this disparity, the study meticulously reports its findings, incorporating the respective prevalence rates. The inclusion of these prevalence rates in medical research serves to clarify the direct benefit to the patient. While the impact of disease prevalence on the accuracy of a diagnostic test has long been recognized in medicine [33], this is to the best of our knowledge often neglected in papers that introduce predictive deep learning methods in medicine. Nonetheless, the prevalence of predictive models is not always the population data, but it could involve only patients, who have already some severe risk factors and therefore it can be difficult to quantify the actual prevalence for some predictive models. While many AI models have the potential to positively impact healthcare, it is crucial to align the research with patients’ interests, as emphasized in [22]. The PPV value gives the direct response to the patient how likely the diagnosis is correct. In cases of low prevalence, this accuracy level can significantly influence the PPV and, consequently, the relevance to patients, as illustrated in Fig. 4. Therefore, the predictive metrics need to be considered carefully, when applying this method. It could be used in a pre-screening to evaluate the risk before considering an image-based analysis or use it in a multi modular deep learning method.

This discussion aims not to discourage the utilization of AI, especially with the introduction of innovative architectures as demonstrated in [34], which represent crucial steps towards a future of more fully automated medical applications. However, it serves as a call for the research community to provide a broader context when introducing predictive models for medical benefits.

### Future work

Further work in predictive models for detecting intracranial stenosis should be according to the way clinicians conduct diagnosis by considering data from multiple sources to reach a conclusion. Therefore, the classification and detection of MRA should be based on multi modular deep learning methods to leverage all the data for highest diagnostic performance. To realize this, a possibility is to merge the feature space of the images with the demographic data via multi head attention layers.

### Strengths and limitations

Our predictive model used simple demographic data and blood lipids to determine ICAS and achieved higher classification results compared to detection algorithms[14]. While detection algorithms use costly TOF-MRAs, which need extensive time and money. Nonetheless, the model was limited on classification, and it was not possible to detect or conclude the severity of the intracranial stenosis. Furthermore, due to the low prevalence of the condition, the PPV value was too low for the model to have clinical relevance.

## Conclusion

In summary, our investigation has demonstrated that the incorporation of risk factors derived from clinical and demographic data yields a predictive accuracy of 81%, surpassing the classification of current TOF-MRA detection algorithms [14]. If the demographic data has low correlation with the images, adding this information should enhance the accuracy of an image-based classification algorithm. Furthermore, the clinical relevance of this accuracy is questionable, as evidenced by a low positive predictive value (PPV) of 19%. Emphasizing the significance of prevalence as a case study, we underscore the importance of considering this parameter for assessing clinical relevance, particularly in light of prevalent trends in medical predictive model publications that predominantly focus on reporting accuracy alone [35–37].

## Electronic supplementary material

Below is the link to the electronic supplementary material.

Supplementary Material 1

## Data Availability

The data used in the analysis is not freely available, but may be obtained via an application to the Tromsø Study (tromsous@uit.no).
