# Supplementary material for: Intracranial stenosis prediction using a small set of risk factors in the Tromsø Study
Source: BMC Med Inform Decis Mak. 2025 Feb 20;25:95. doi: 10.1186/s12911-025-02896-x (PMC11843764; doi:10.1186/s12911-025-02896-x)
Supplement: Supplementary file 1 — Supplementary Material 1 [file 12911_2025_2896_MOESM1_ESM.docx]

Supplementary Material


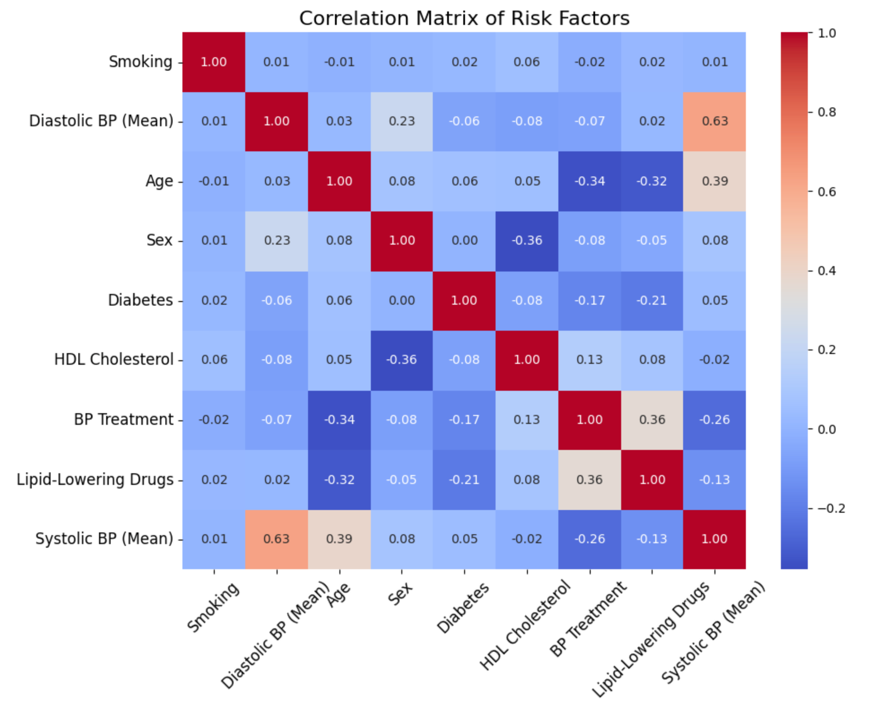


Figure 1 Correlation Matrix for Risk factors used in the study.


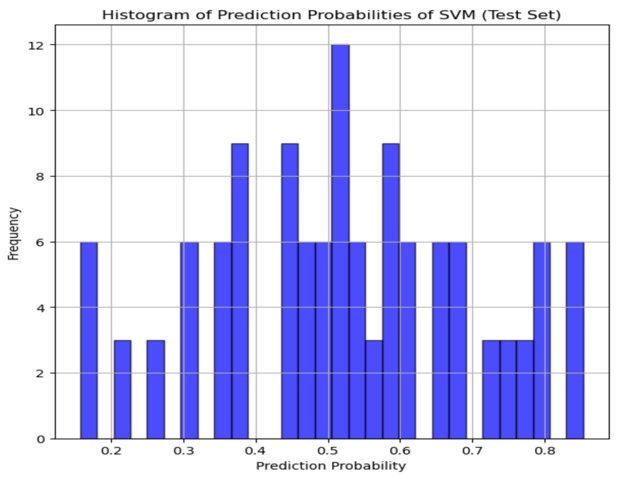

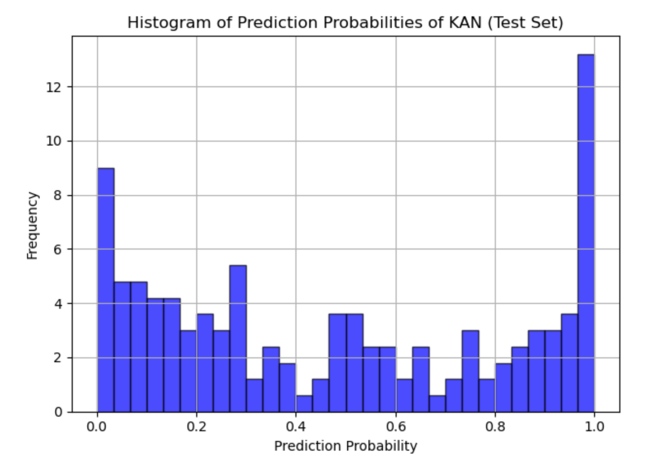

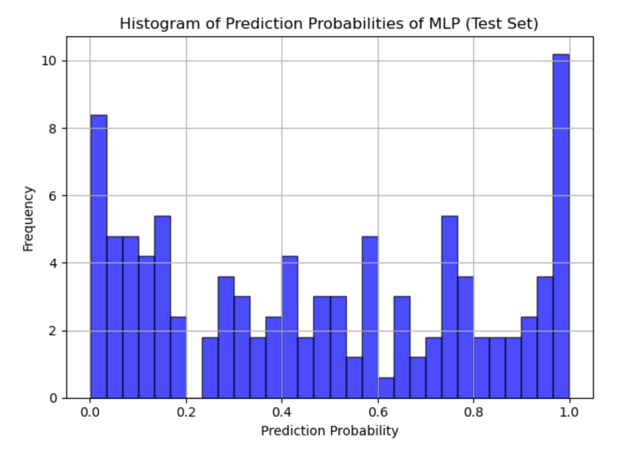


Figure 2 Histogram of probabilities of test set predictions.
